# Health related quality of life among people with mental illness: The role of socio-clinical characteristics and level of functional disability

**DOI:** 10.3389/fpubh.2023.1134032

**Published:** 2023-02-16

**Authors:** Semira Defar, Yacob Abraham, Yared Reta, Bedilu Deribe, Meskerem Jisso, Tomas Yeheyis, Kurabachew Mengistu Kebede, Bereket Beyene, Mohammed Ayalew

**Affiliations:** ^1^Department of Midwifery, College of Medicine and Health Science, Hawassa University, Hawassa, Ethiopia; ^2^School of Nursing, College of Medicine and Health Science, Hawassa University, Hawassa, Ethiopia; ^3^School of Public Health, College of Medicine and Health Science, Hawassa University, Hawassa, Ethiopia; ^4^Department of Anesthesia, Faculty of Medicine, College of Medicine and Health Science, Hawassa University, Hawassa, Ethiopia

**Keywords:** health related quality of life, functional disability, predictors, mental illness, SF-12

## Abstract

**Background:**

People with mental illness (PWMI) have declining health related quality of life (HRQoL), which is frequently equivalent to or greater than that of medical disorders. Although, HRQoL is rapidly being recognized as an essential treatment outcome indicator in modern psychiatry, research on the identification and significance of factors impacting QoL in PWMI is still in its early stages.

**Objective:**

The aim of this study was to identify predictors of HRQoL among people with mental illness who underwent outpatient follow-up in Sidama region, southern Ethiopia.

**Methods:**

We conducted a multicenter, cross-sectional study from April-1, to May-30, 2022. A total of 412 participants took part in the study, using an interviewer-administered structured questionnaire. The HRQoL was measured using the 12-item Short-Form Health Survey-Version 2 (SF-12v2) scale. To describe different variables, descriptive statistics were employed. To find independent HRQoL predictors, we used multivariable linear regression analysis. *P*-value of <0.05 were declared statistically significant at 95% confidence interval (CI).

**Result:**

Out of 412 participants, nearly two-third 261 (63.3%) were male and nearly half 203 (49.3%) were diagnosed as schizophrenia. HRQoL was positively associated with social support (β = 0.321) and being single (β = 2.680). Conversely, functional disability (β = −0.545), being a student (β = −4.645) and jobless (β = −3.279) by occupation, and being diagnosed with depression (β = −2.839) were negatively impacted HRQoL among PWMI.

**Conclusion:**

HRQoL of people with mental disorders in this study was significantly associated to social support, marital status, occupation, diagnosis and level of functional disability. Therefore, the mental health care system should develop HRQoL promoting measures that enhance PWMI functioning, social support and employment.

## Introduction

Mental illness (MI) is a term used to describe a range of mental health disorders characterized by mental, behavioral, or emotional impairments that have a significant impact on the functionality, major life activities, and quality of life of those suffering from the condition (1). Globally, the rise in overall morbidity and disability is mostly fueled by mental disorders (2, 3). Overtaking its other primary causes, such as non-communicable diseases and injuries, psychiatric disorders account for one-third (32.4%) of the years lived with disability burden (4). Depression, anxiety disorders, schizophrenia, and bipolar disorder are the most frequent mental disorders that lead to years lived with disability (YLDs) (5).

Mental illness is the most prevalent non-communicable disease in Ethiopia. Indeed, mental illness accounted for 11% of the total disease burden in a primarily rural area of Ethiopia, with schizophrenia and depression ranking among the top 10 highest burdens (6). According to a recent systematic review and meta-analysis, the prevalence of common mental illness is 21.6% in the general population and 36.4% in patients with co-morbid conditions (7). Rapidly spreading communicable diseases (like HIV and TB), non-communicable chronic diseases, unintended teenage pregnancies, malnutrition, and insecurity, including violence, may be intertwined with the rapidly rising pattern of these mental health issues in the country (7). Patients with severe mental illnesses have a lower quality of life than the general population (8), because their mental illness makes it harder for them to accomplish many of their daily tasks, reducing their level of independence and resulting low self-confidence and self-esteem.

Quality of life (QoL) and health-related quality of life (HRQoL) are terms that are frequently used interchangeably (9). QoL refers to an individual's subjective wellbeing in relation to their health, psychological condition, beliefs, interpersonal relationships, and relationship to significant environmental conditions such as living condition, security, accessibility to medical care, opportunity for recreation, and facilities (10). Furthermore, HRQoL is defined as a person's perceived wellbeing in physical, mental, and social domains of health, as well as how well they function in their daily lives (11). HRQoL is an important disease indicator in health care HRQoL can also be utilized to examine disease severity, treatment outcomes, client satisfaction with health care, effectiveness of healthcare, patients' overall wellbeing, and the expense of a specific intervention (12).

In recent years, QoL has been recognized as an important measure of disease impact among PWMI (8), and it has been shown to be considerably impaired in these individuals (13). Improving the HRQoL of PWMI in the community has also become an important public health agenda. Integration of social, psychological, and medical care is required to improve HRQoL (14, 15). PWMI have declining HRQOL, which is frequently equivalent to or greater than that of medical disorders (16). This has a substantial impact on treatment adherence, relapse rates, ability to engage in and/or enjoy social and vocational activities, future prospects, and medical issues (17).

A decline in HRQoL in individuals with mental illness has been demonstrated in several studies to be clinically significant. Socio-demographic variables like age, sex, marital status, living condition (18, 19), and low level of education (20) explained the variation in HRQoL in PWMI. Clinical factors such as being depressed, having positive and negative symptoms in schizophrenia, poor social support (21) and greater disability (20) predicted poor HRQoL in PWMI. Social support has been shown to boost self-esteem, coping abilities, and resilience while also minimizing stigma (22). A recent study also revealed that severity of symptoms, duration of treatment and number of hospitalization determines HRQoL among patients with schizophrenia and depression (23). Likewise, recent studies in Ethiopia elicited that age of onset of illness, perceived stigma, medication compliance, substance use, and comorbid medical illness are significantly impacted HRQoL among PWMI (24–26).

Severe mental illnesses may cause functional disability (27–30), that can significantly interfere with daily life activities, negatively impact health outcomes, and have a significant impact on patients QoL. The HRQoL of people with mental illness may be negatively impacted by functional restrictions brought on by a disability associated with mental illness (31).

Although, HRQoL is rapidly being recognized as an essential outcome in modern psychiatry, research on the relative impact of the co-occurrence of mental disorders is still in its early stages. The identification and significance of factors impacting HRQoL in PWMI is of special relevance because HRQoL is more frequently used as a patient-related outcome in research. Thus, the current study would look at the quality of life of those suffering from PWMI in southern Ethiopia. The purpose of this study was to fill the gap in the local literature by investigating the potential of accessible variables, specifically demographic and clinical parameters, and disability due to mental illness to predict HRQoL in patients with mental illness.

## Materials and methods

### Description of the study area

Institution based cross sectional study was conducted between April-1, and May-30, 2022 among patients with mental illnesses in selected hospitals in Sidama national regional state (Hawassa university comprehensive specialized hospital, Adare general hospital, and Yirgalem general hospital).

### Study participants

Four hundred and twelve participants were consecutively recruited from psychiatry outpatient clinics at the three selected hospitals in Sidama regional state, southern Ethiopia. Three hospitals (one specialized tertiary and two general hospitals) were chosen to provide outpatient services to those suffering from mental illnesses in the region and adjoining regions of the southern Nation Nationalities and People Region and Oromia. Eligibility requirements included a clinical diagnosis of mental disorders, being 18 or older, and being in a stable mental condition at the time of examination. To assess the current intensity of symptoms, the Clinical Global Impression Severity (CGI-S) Scale with a seven-point scale was utilized. The CGI-S scale spans from 1 (normal) to 7 (very unwell) (32). Patients with a CGI-S score of 6 or higher were excluded from the study because the intensity of their symptoms may prevent them from responding appropriately.

### Instruments

The data were collected using an interviewer-administered structured questionnaire. The questionnaire contains sociodemographic factors, clinical related factors, social support, disability related items, and health related HRQoL items. Some of the clinical factors such as clinical diagnosis, duration of illness, substance use history, and comorbidities were obtained from the patient's chart. Clinical diagnosis of mental disorders were made by psychiatrists, psychiatry nurses, and senior or expert mental health professionals based on the Diagnostic Statistical Manual for Mental Disorders-5 (DSM-5) diagnostic criteria.

Level of social support among patients with mental illness was assessed using the 3-item Oslo social support scale (OSSS) and the scores range from 3 to 14. It is categorized as poor [3–8], moderate [9–11], and strong [12–14] social support (33), and a higher score indicates better social support. In the previous study (24), it had a good internal consistency (Cronbach's alpha) of 0.93, and in the current study, it had an acceptable Cronbach's alpha of 0.75.

The World Health Organization Disability Assessment Schedule (WHODAS-2.0) 12-item version was used to assess functional disability. The WHODAS is a cross-cultural measure of a person's activity limitations and social restrictions in the last 30 days that is not condition specific (34). A number of research have established the WHODAS's psychometric properties (35, 36). The WHODAS has also been developed and validated in a variety of languages and cultures, including rural Ethiopia (37). In the current study, it had a very good Cronbach's alpha of 0.94.

To assess quality of life (QoL), the 12-item Short-Form Health Survey-Version 2 (SF-12v2) scale was used. It was designed to assess multidimensional elements of physical and mental health in the general population as well as people suffering from chronic conditions such as mental disorders, and a higher score indicates better self-reported QoL (38). The scale has eight (physical role, physical functioning, general health, body pain, social functioning, vitality, mental health, and emotional role) subdomains. Furthermore, two general scores were computed: the physical health component summary (PHCS) and the mental health component summary (MHCS), with higher score indicating greater QoL (39). Thus, SF-12 appears to be a reliable psychometric instrument for assessing HRQoL in PWMI (40). It has been validated and developed in varieties of settings and cultures with good psychometric properties (41–46). It had a very good Cronbach's alpha of 0.92 in the current study.

### Data analysis

Collected data were entered to Epi-data version 3.1 and exported to SPSS version 24 for windows for analysis. Descriptive statistics such as frequency, percentage, mean, standard deviation, and median were used to describe different variables. Assumptions such as normality, lack of multi-collinearity among explanatory variables, presence of linearity relationship, independence and homoscedasticity of the errors were checked. Simple and multivariable linear regression were done to identify independent predictors of QoL among patients with mental illness. *P*-value of < 0.05 were declared statistically significant at 95% confidence interval (CI).

## Results

### Socio-demographic characteristics of study participants

A total of 412 participants involved in the study. Nearly two-third 261 (63.3%) participants were male. About one-third 147 (35.7%) participants were at age group 18–27 years, 215 (52.2%) were single and 245 (59.5%) were protestant by religion (Table 1).

**Table 1:** Socio-demographic characteristics of study participants to HRQoL among people with mental illness in Sidama region, south Ethiopia, 2022 (*n* = 412).

**Variable**	**Categories**	**Frequency**	**Percentage (%)**
Age	18–27 years	147	35.7
	28–37 years	128	31.1
	38–47 years	78	18.9
	≥48 years	59	14.3
Sex	Male	261	63.3
	Female	151	36.7
Marital status	Single	215	52.2
	Married	179	43.4
	Divorced	18	4.4
Religion	Protestant	245	59.5
	Orthodox	110	26.7
	Muslim	38	9.2
	Others	19	4.6
Educational status	Illiterate	34	8.3
	Primary	129	31.3
	Secondary	122	29.6
	College and above	127	30.8
Occupation	Gov't employee	74	18.0
	Farmer	55	13.3
	House wife	55	13.3
	Student	69	16.7
	Merchant	45	10.9
	Daily labor	24	5.8
	Jobless	77	18.7
	Other	13	3.2
Place of residence	Rural	163	39.6
	Urban	249	60.4
Living with	Family	375	91.0
	Alone	30	7.3
	Others	7	1.7
Time taken to hospital with any means of transport	< 60 min	196	47.6
	60–180 min	154	37.4
	≥180 min	62	15.0

### Clinical characteristics of study participants

Among the 412 participants, 161 (39.1%) reported the onset of their illness between the ages of 21 and 30 years. Furthermore, 158 (38.3%) had been ill for 1 year or less, about half 219 (53.2%) had a history of suicidal ideation, 120 (29.1%) had a lifetime history of substance use, and nearly half 203 (49.3%) were diagnosed with schizophrenia, as shown in Table 2.

**Table 2:** Clinical characteristics of study participants to health related quality of life among people with mental illness in Sidama region, south Ethiopia, 2022 (*n* = 412).

**Variable**	**Categories**	**Frequency**	**Percentage (%)**
Age at first onset of illness	< 20 years	114	27.7
	21–30 years	161	39.1
	≥31 years	137	33.3
Duration of illness	≤ 12 months	158	38.3
	13–60 months	110	26.7
	≥61 months	144	35.0
Suicidal ideation	Yes	219	53.2
	No	193	46.8
Suicidal attempt	Yes	139	33.7
	No	273	66.3
Life time substance use	Yes	120	29.1
	No	292	70.9
Last 1 year substance use	Yes	76	18.4
	No	44	10.7
Type of substance	Cigarette	11	2.7
	Khat	32	7.8
	Alcohol	26	6.3
	Others	8	1.9
DSM-5 diagnosis	Schizophrenia	203	49.3
	Other psychotic disorders	75	18.2
	Depressive disorders	81	19.7
	Bipolar disorders	28	6.8
	Anxiety disorders	23	5.6
	Others	2	0.5
Family history of mental illness	Yes	45	10.9
	No	367	89.1
Comorbid physical disease	Yes	100	24.3
	No	312	75.7
Mean social support score		9.62 ± 2.59	
Mean functional disability (WHODAS) score		10.48 ± 10.93	

### Health related quality of life score

The mean score of overall SF-12 (Health related quality of life) was 38.54 ± 10.33. Whereas, the mean physical health component and mental health component score were found to be 17.15 ± 4.37 and 21.38 ± 6.63, respectively as shown in Table 3.

**Table 3:** Descriptive statistics of domains of health related quality of life among people with mental illness in Sidama region, south Ethiopia, 2022 (*n* = 412).

**Domain**	**Mean ±SD**	**Median**	**Mode**	**Range**	**Minimum**	**Maximum**
General health	2.93 ±1.07	3.00	3	4	1	5
Physical functioning	4.74 ±1.32	5.00	6	4	2	6
Role physical	6.10 ±1.94	6.00	6	8	2	10
Role emotional	6.72 ±2.29	6.50	6	8	2	10
Bodily pain	3.39 ±1.31	3.00	5	4	1	5
Mental health	7.30 ±2.57	8.00	10	8	2	10
Vitality	3.58 ±1.35	4.00	5	4	1	5
Social functioning	3.39 ±1.32	4.00	5	4	1	5
Physical health component	17.15 ± 4.37	18.00	19	20	6	26
Mental health component	21.38 ± 6.63	22.00	30	24	6	30
Overall quality of life	38.54 ±10.33	39.00	33	44	12	56

### Independent predictors of HRQoL

After controlling for confounding variables, we identified five independently significant predictors for HRQoL among PWMI. Those PWMI who had high social support scale score were better HRQoL (β = 0.321; 95% CI = 0.003, 0.639; *p* = 0.048). Functional disability (WHODAS-score) had significant negative impact on HRQoL among PWMI (β = −0.545; 95% CI = −0.620, −0.470; *p* = < 0.001). PWMI who were single had better HRQoL as compared to married individuals (β = 2.680; 95% CI = 0.571, 4.789; *p* = 0.013). Patients who were student (β = −4.645; 95% CI = −7.865, −1.424; *p* = < 0.001) and jobless (β = −3.279; 95% CI = −6.193, −0.365; *p* = 0.028) by occupation had lower HRQoL scores as compared to employed patients. In addition, patients with depression were lower HRQoL score as compared to patients with schizophrenia (β = −2.839; 95% CI = −5.110, −0.568; *p* = 0.014) as described in Table 4.

**Table 4:** Simple and multiple linear regression for health related quality of life among patients with mental illness in Sidama regional state, southern Ethiopia, 2022 (*n* = 412).

**Variables**		**Simple linear regression**	**Multiple linear regression^†^**
		β **(95% CI)**	β **(95% CI)**
Age (years)		0.018 (−0.066, 0.102)	0.024 (−0.065, 0.113)
Time taken to hospital (hours)		0.142 (−0.293, 0.576)	0.100 (−0.263, 0.463)
Delay treatment seeking (weeks)		−0.060 (−0.116, −0.005)^*^	−0.024 (−0.070, 0.023)
Duration of illness (weeks)		0.003 (−0.011, 0.016)	0.005 (−0.007, 0.017)
Social support score		0.607 (0.225, 0.989)^**^	**0.321 (0.003, 0.639)** ^***^
Functional disability score		−0.575 (−0.648, −0.502)^***^	**−0.545 (−0.620**, −0.470)^*^
Sex	Male	0	
	Female	0.417 (−1.611, 2.495)	0.327 (−1.903, 2.557)
Marital status	Married	0	
	Single	−0.566 (−2.616, 1.485)	**2.680 (0.571, 4.789)** ^***^
	Divorced	−4.711 (−9.723, 0.300)	−1.253 (−5.701, 3.195)
Educational status	Illiterate	1.081 (−2.852, 5.014)	0.469 (−3.307, 4.245)
	Primary	0.398 (−2.148, 2.944)	0.700 (−1.738, 3.137)
	Secondary	0.003 (−2.585, 2.579)	0.488 (−1.738, 3.137)
	Tertiary	0	
Occupation	Gov't employee	0	
	Farmer	−0.342 (−3.887, 3.203)	−0.099 (−3.559, 3.362)
	Housewife	−0.160 (−3.705, 3.385)	0.923 (−2.673, 4.520)
	Student	−5.442 (−8.774, −2.109)^**^	**−4.645 (−7.865**, −1.424)^**^
	Merchant	−0.728 (−4.492, 3.037)	−0.849 (−4.166, 2.468)
	Daily labor	−3.155 (−7.833, 1.522)	−2.740 (−6.839, 1.359)
	Jobless	−5.269 (−8.511, −2.027)^**^	**−3.279 (−6.193**, −0.365)^***^
	Others	−4.290 (−10.278, 1.698)	0.930 (−4.062, 5.921)
Residence	Rural	3.120 (1.095, 5.146)^**^	1.135 (–.778, 3.049)
	Urban	0	
Living status	Alone	1.488 (−2.012, 4.988)	−0.481 (−3.661, 2.699)
	With family	0	
Diagnosis	Schizophrenia	0	
	Other psychotic	−2.301 (−5.029, 0.428)	−0.715 (−3.044, 1.614)
	Depression	−3.657 (−6.311, −1.003)	**−2.839 (−5.110**, −0.568)^***^
	Bipolar	−1.584 (−5.654, 2.487)^**^	−1.141 (−4.549, 2.266)
	Anxiety	−2.501 (−6.781, 1.779)	−0.596 (−4.263, 3.070)
Comorbid diagnosis	Yes	−2.357 (−5.559, 0.846)	0.380 (−2.258, 3.019)
	No	0	
Substance use	Yes	−1.590 (−3.789, 0.609)	−1.879 (−3.862, 0.104)
	No	0	

^*^p-value < 0.001; ^**^p-value < 0.01; ^***^p-value < 0.05.

^†^Multiple linear regression model reported that F_(27, 384)_ = 11.111, p < 0.001, with R^2^ = 0.439. The bold values indicate statistically significant variables.

## Discussion

We evaluated all patients with a diagnosis of mental disorders who attended follow-up at public hospitals in Sidama National Regional State in the current study. Taking into account the variety of mental illness outcomes (47, 48), sociodemographic, general clinical characteristics and functional impairment, as well as health related quality of life, were evaluated in order to confirm the association between these variables and health related quality of life. Hence, good social support and being single was positively associated with QoL, however, functional disability, being student and jobless by occupation, and diagnosed with depression were negatively affect HRQoL in PWMI.

Regarding the association of social support and QoL, we found a statistically significant association between OSSS and HRQoL. In the current study, a higher social support score among those with mental illness is a significant positive predictor of a greater HRQoL. This finding is supported by other studies done in Ethiopia (24) and other parts of the world (49–52). Cohen et al., discovered that the absence of loneliness and consistent social interactions predicted subjective wellbeing in people with severe mental illness such as schizophrenia (53). Poor social support is associated with depression and non-compliance to treatment (54, 55). On other hand, adequate social support has been shown to boost resilience, coping mechanisms, self-esteem, lowering stigma and improving life satisfaction (22, 56). As a result, social support may increase HRQoL by buffering stress, lowering depression, and boosting the adaption process (57).

Similar to previous studies (58, 59), in this study, the HRQoL of people with mental illness was negatively associated to their level of functioning/disability status, which might be represented in low employment rates and social interactions. Researchers have discovered that a worse HRQoL is connected with the incapacity to perform functional life tasks, such as activities of daily living such as bathing, dressing and instrumental activities of daily living like driving, budgeting (31, 60). Disability associated with mental illness can cause impairments, limitations, and restrictions to activities and participation in relation to the environment which may compromise HRQoL (59). This suggests that HRQoL and functioning can be employed as essential parameters in the creation of mental healthcare strategies, health condition monitoring, and social reintegration of these persons, hence helping the introduction of new assisted living facilities and enhancing the existing one. Furthermore, finding the consequences of the condition is important for choosing more effective treatments and determining the best strategies for public resource allocation. The functioning assessment and limitations of the person with mental disorders play a vital role in this process (61).

We found out that being single increases HRQoL scores as compared to married PWMI. Similar findings in earlier study reported that single women and single participants 30–39 years old had higher HRQoL (62). The fact that being married increases societal burdens such as shouldering family responsibilities could explain why single PWMI had greater HRQoL in our study. As a result, married individuals may have a negative perspective of their HRQoL, such as feeling like a burden to their families, failing to care for children or other family members, and failing to appropriately handle duty. Furthermore, problems among married people are related to the partner, such as challenging marital intimacy and commonality, restructuring of familial and partnership responsibilities, and reinterpretation of mutual life plans (63). However, other reports also indicated that being married predicts better HRQoL (64). That is, those who have a partner are more likely to express their emotions, daily experiences, and thoughts, as well as receive enough social support, resulting in a good outlook on life.

In this study we found that students and jobless patients have lower HRQoL score than the employed patients. Similarly, other studies have found that employed patients have higher HRQoL scores than those who are unemployed (65–68). This could be explained by higher self-esteem among patients with employment, which has been described as a moderating factor between employment and HRQoL, as well as having a larger social network as a result of employment (69, 70). Moreover, the relationship between employment status and HRQoL in our study may be due to differences in income because employed patients had higher source of income while students and unemployed patients may not have had enough income to cover their basic needs.

We also found that patients diagnosed with depression have decreased HRQoL than patients with schizophrenia. Similarly, depression has been identified as a critical predictor of subjective HRQoL in prior studies (68, 71, 72). Also, low HRQoL has been associated to depression among outpatients in Spanish and French studies (73, 74). This is due to the fact that patients suffering from psychosis were unaware of their condition and social milieu. Individuals with depression, on the other hand, have symptoms such as anhedonia, depressed mood, pessimistic outlook, decreased motivation and energy level, and are thus more likely to report poor HRQoL than patients with schizophrenia (75). Therefore, the possibility that depressed individuals may have unfavorable perceptions of their circumstances, could have a detrimental impact on how they perceive their HRQoL.

The following limitations should be kept in mind in this study. Firstly, this study only used cross-sectional data. Therefore, no causal relationships could be identified. Secondly, HRQoL and disability were self-reported, which might be prone to social desirability bias. Thirdly, some clinical factors like treatment adherence, adverse drug reactions, and the types of medications the participants had taken were not addressed in this study. Lastly, our results might only apply to people who are actively engaged in treatment. Thus, the results of this research may not accurately reflect the experiences of PWMI who do not actively involved in modern psychiatry care. Therefore, additional research is required to confirm and broaden the findings in various individuals with mental disorders, including those PWMI outside the formal mental healthcare system.

## Conclusion

In conclusion, the HRQoL of people with mental disorders in this study was significantly associated to their social support, marital status, occupation, diagnosis and level of disability due to their mental illness. Taking into account all of these variables, the mental health care system should develop HRQoL promoting measures that enhance PWMI functioning, social support and employment.

## Data availability statement

The original contributions presented in the study are included in the article/supplementary material, further inquiries can be directed to the corresponding author.

## Ethics statement

The studies involving human participants were reviewed and approved by Hawassa University, College of Medicine and Health Sciences, Institutional Review Board (IRB) with reference number: IRB/030/14. The participants provided their written informed consent to participate in this study. This study complies with the Declaration of Helsinki.

## Author contributions

SD, MA, BD, YA, YR, and MJ participated in the conception and designed the study and involved in the data collection. SD, MA, YA, TY, KK, BB, and MJ did the analysis of the study. SD, MA, and MJ prepared the manuscript for publication. BD, YA, YR, TY, KK, and BB critically reviewed the manuscript. All authors read and approved the final manuscript.
